# Investigation of the antimicrobial properties of nanoclay and chitosan based nanocomposite on the microbial characteristics of Gouda cheese

**Published:** 2020-04

**Authors:** Mojtaba Mohammadzadeh-Vazifeh, Seyed Masoud Hosseini, Ali Mohammadi, Mahdi Jahanfar, Hadi Maleki

**Affiliations:** 1 Department of Microbiology and Microbial Biotechnology, Faculty of Life Sciences and Biotechnology, Shahid Beheshti University, Tehran, Iran; 2 Department of Microbiology, Faculty of Biological Sciences, Alzahra University, Tehran, Iran; 3 Department of Cell and Molecular Biology, Faculty of Life Sciences and Biotechnology, Shahid Beheshti University, Tehran, Iran

**Keywords:** Gouda cheese, Nanoclay, Chitosan, Nanocomposite, Antimicrobial property

## Abstract

**Background and Objectives:**

In recent years, active packaging has been introduced as a new method to better preserve food. Chitosan and nanoclay have been used for preparation of an active nanocomposite with respect to their antimicrobial properties to investigate its effects on the microbial limitation in Gouda cheese.

**Materials and Methods:**

Nanoclay film, chitosan film, chitosan-based nanocomposites and nanoclay-based nanocomposites were prepared and their antimicrobial properties were evaluated to the microbial limitations of Gouda cheese consist of coliforms, *Escherichia coli, Salmonella* spp., coagulase-positive *Staphylococcus*, mold and yeast by agar diffusion method.

**Results:**

The results indicated, the best antimicrobial effect belonged to nanocomposite film with the composition of chitosan 3 wt% by adding nanoclay 1 wt%, which can prevent microbial characteristics of Gouda cheese.

**Conclusion:**

The chitosan and nanoclay nanocomposite had excellent antibacterial activity and performed well against microbial limitations (coliforms, *E. coli, Salmonella* spp., coagulase-positive *Staphylococcus*, mold and yeast) of Gouda cheese. Therefore, the nanocomposite may be possibly used as a surface coating in addition to Gouda cheese as well as similar cheeses and other food to enhance microbial characteristics and extend shelf life.

## INTRODUCTION

Packaging materials have the protective ingredients to increases the shelf life of food by preparing good antimicrobial, chemical and physical properties. Packaging made from biopolymers can be a barrier to moisture, water vapor, and gases and it has a good potential for adding materials such as anti-fungal, antioxidants and antimicrobials compounds, colors, and other food components (1, 2)

Highly biodegradable biopolymers derived from renewable sources of agriculture are suitable alternatives in this regard. Polysaccharides, proteins, fats or a mixture of them are used to produce biodegradable nanocomposites (3, 4).

Chitosan is a linear polysaccharide derived from acetylation of chitin, found in the external skeleton of arthropods such as insects, crabs, shrimp, lobster and cell wall, a particular type of algae. This is the most abundant polysaccharide in nature. Chitosan is an active biomolecule that has a high potential for food packaging due to its biodegradability, biocompatibility, antimicrobial activity, non-toxicity, and film-forming capacity (1, 5, 6).

Chitosan has several advantages over other types of disinfectants and synthetic polymers such as biodegradability, gas barrier for increasing shelf life, and antimicrobial for increasing of spoilage time. The use of biopolymer films has some limitations and problems with their performance such as inherent water sensitivity and relatively low resistance, especially in wet environments. Chitosan is no exception to this general feature, and its hydrophilic properties and consequently its poor mechanical properties in wet environments and the insufficient thermal and toughness resistance have made it difficult to use in the packaging industry (1, 5, 6).

The nanoclay (montmorillonite) consists of sheets of one nanometer thick and a length of 100 to 500 nm, and as a filler, it enhances the properties of polymers. Since the clay layer creates a barrier against the influence of gases and vapor (by creating a grubbed area), its addition to biological polymers effectively increases their deterioration properties (1, 3, 7).

Gouda cheese is one of the most popular cheeses worldwide. It is a semi-hard to hard cow’s milk cheese introduced for the first time by the Netherlands. When Gouda is aged the cheese changes its texture with a strong flavor, and with a sharp yet sweet taste at the same time. In the process for making Gouda cheese, a lot of microbial and chemical changing occurs. At the final stage of production, it is waxed with paraffin-based ingredients and it dried over time until arrives. After the cheese ripening it would deliver to the market (8, 9).

There are well published International and National Standards on Gouda cheese that describe the sensory, microbial, chemical, physical and packaging properties of this type of cheese. These standards are defined for the microbial acceptance level for coliforms, *E. coli, Salmonella* spp., coagulase-positive *Staphylococcus*, mold and yeast (Table 1) (10, 11).

**Table 1.** Examined microbial acceptance level of Gouda cheese

**Microorganisms**	**Colony forming Unit (CFU/g)**
Coliforms	<10
*Escherichia coli*	Negative
*Salmonella*	Negative
Mold and yeast	<10^2^
Coagulase-positive	Negative
*Staphylococcus*	

This study aimed to improve the antimicrobial properties and inhibitory effects of chitosan by adding clay nanoparticles to polymer matrices and determining the best uniform dispersion of nanoclay and chitosan and forming nanocomposites to optimize them for use against microbial limitations of Gouda cheese.

## MATERIALS AND METHODS

### Materials.

Chitosan was obtained from Sigma-Aldrich (Cat. No. 448869) with 75–85% degree of acetylation and low molecular weight, also, the Montmorillonite nanoclay and acetic acid were obtained from Sigma-Aldrich (Cat. No. 69866) and Merck (Cat. No. 100063) respectively.

Salmonella-Shigella (SS) Agar medium for *Salmonella* spp., Mannitol salt agar for *S. aureus*, Violet Red Bile Lactose Agar for coliforms, Lauryl Sulfate Broth and MacConkey agar for the examination of *E. coli* and Yeast Extract Glucose Chloramphenicol Agar for mold and yeast obtained from Merck (12).

The standard microorganisms were supplied by the Ibresco Co, (Iran) comprised *E. coli* (strain ATCC 19118), *S. aureus* (ATCC 6538), *S. cerevisiae* (PTCC 5074), *A. brasiliensis* and *S. enterica* (PTCC 1709).

### Preparation of nanocomposite.

A designated amount of chitosan powder (1, 3 and 5 wt%) was dissolved in 1% v/v acetic acid solution at 90 °C for 20 min in order to obtain 1, 3 and 5% w/v aqueous chitosan solution using a magnetic stirring at 322 g. Then the solution was cooled down to room temperature (18–25°C) for further process (1, 3, 13).

Three different concentrations of nanoclay suspensions (1, 3 and 5 wt%) were made by diffusing appropriate amounts of nanoclay into 10 mL of 1% v/v acetic acid solution for 24 hours using Ultrasonic Homogenizer. (Hielscher, UP400St, Germany) (1, 3, 13).

Afterward, 3 different solutions of chitosan were added slowly into the 3 different concentrations pretreated nanoclay suspensions. Also, 3 different solutions of nanoclay were added slowly into the 3 different concentrations pretreated by chitosan suspensions.

After removal of bubbles by degassing under vacuum for 5 min, 30 mL of each mixture were cast evenly onto glass petri dishes (12 cm in diameter), and dried in an oven at 30 °C for 24 h.

There were 9 samples of chitosan-based nanocomposites containing various amounts of nanoclay and 9 samples of nanoclay-based nanocomposites containing various amounts of chitosan. Also, pure chitosan without any nano reinforcements and pure nanoclay without any nano reinforcements were used as a control (Tables 2, 3 and 4).

**Table 2.** Samples of chitosan based nanocomposites

**Samples**	**Chitosan (% w/v)**	**Nanoclay (% w/v)**
1	1	1
2	1	3
3	1	5
4	3	1
5	3	3
6	3	5
7	5	1
8	5	3
9	5	5

**Table 3.** Samples of nanoclay based nanocomposites

**Samples**	**Nanoclay (% w/v)**	**Chitosan (% w/v)**
1	1	1
2	1	3
3	1	5
4	3	1
5	3	3
6	3	5
7	5	1
8	5	3
9	5	5

**Table 4.** Control samples

**Samples**	**Nanoclay (wt%)**	**Chitosan (wt%)**
1	1	0
2	3	0
3	5	0
4	0	1
5	0	3
6	0	5

Dried nanocomposites were then peeled off, and preconditioned at 25 °C and 50% RH for at least 48 h in a desiccator containing saturated magnesium nitrate solution before further evaluation) (1, 3, 13).

### Method of microbial enumeration.

In refer to Codex C-5; 2013, and ISIRI (Institute of Standards and Industrial Research of Iran) 9013 and 2406 standards, all collected samples examined for microbial enumeration of coliforms, mold and yeast, as well as detection of *E. coli, Salmonella* and coagulase-positive *Staphylococcus* (10, 11, 12). In brief, enumeration of coliforms according to ISO 4832, *E. coli* according to ISO 11866-1, *Salmonella* according to ISO 6785 and coagulase-positive *Staphylococcus* according to ISO 6888 – 3 and mold and yeast according to ISO 6611 standards tested (14–19).

### Antimicrobial activity of films.

The agar diffusion method was used to determine the antimicrobial activity of the nanocomposites films (20).

Each bacteria containing *E. coli* (strain ATCC 19118), *S. aureus* (ATCC 6538), *S. cerevisiae* (PTCC 5074), *A. brasiliensis* and *S. enterica* (PTCC 1709) added into 10 mm Brain heart infusion broth (BHI Broth) using the sterile loop and broth tubes incubated at 37 °C and 25 °C for 24 h and 48 h. Then the tubes were cultured linearly using sterile loops on Nutrient Agar and Sabouraud dextrose agar (SDA) culture medium. The Petri dishes incubated at 37 °C and 25 °C for 24 h and 72 h.

Isolated colonies of the same shape were transferred to a tube containing 5 ml of physiological serum using sterile swabs. Suspensions of turbidity were examined by spectrophotometer at 625 nm. Eventually, 0.5 McFarland was obtained from each microorganism. From each of the suspension tubes was cultured on their media using sterile swabs. Due to the microbial limitations of Gouda cheese, *E. coli, S. aureus, S. enterica, S. cerevisiae* and *A. brasiliensis* cultivated on MacConkey agar, Mannitol salt agar, SS agar and Yeast Extract Glucose Chloramphenicol agar, respectively. *E. coli, S. aureus, S. enterica, S. cerevisiae* and mold and yeast grew up at 37 °C and 25 °C, respectively.

The films were cut to discs with 10 mm diameter. The disks were placed on cultured media under sterile conditions. Also, 4 plates were considered as a control without any film. Then, mold and yeast plates incubated at 25 °C for 72 h and other plates were incubated at 30 °C for 48 h. The diameter of the appearance of inhibitory halo around the hole was considered as an indicator of the antimicrobial activity of the films.

## RESULTS

### Antimicrobial activity of nanocomposites.

The antimicrobial activity of nanoclay film, chitosan film, chitosan-based nanocomposites and nanoclay-based nanocomposites were investigated with *E. coli, S. aureus, S. enterica, S. cerevisiae* and *A. brasiliensis* as test microorganisms.

### The effect of nanoclay concentration films on antimicrobial activity.

Antimicrobial activity of 1, 3 and 5 wt% nanoclay films was examined on the microbial characteristics of Gouda cheese. The results are shown in Table 5. The results indicated that 1 wt% nanoclay showed the most antimicrobial activity than the other 2 samples on the microbial characteristics of Gouda cheese. This film had the most inhibition in Gram-negative bacteria. Only, 1 wt% nanoclay could inhibit the growth of mold and yeast.

**Table 5.** Antimicrobial activity of investigated nanoclay films

**Microorganisms**	**Nanoclay**		

	**1 wt%**	**3 wt%**	**5 wt%**

	**Inhibition zone diameter (mm)**		
*E. coli*	20 ± 0.23^a^	15 ± 0.54^c^	12 ± 0.35^e^
*S. enterica*	18 ± 0.11^b^	12 ± 0.26^e^	NA
*S. aureus*	15 ± 0.34^c^	13 ± 0.17^d^	11 ± 0.25^f^
*A. brasiliensis*	12 ± 0.82^e^	NA	NA
*S. cerevisiae*	11 ± 0.15^f^	NA	NA

The results were reported as mean ± SD. The different letters in each column indicate significant differences.

NA: Not affected

### The effect of chitosan concentration films on antimicrobial activity.

Antimicrobial activity of 1, 3 and 5 wt% chitosan films was tested on the microbial characteristics of Gouda cheese. The results of Table 6 demonstrate that all samples demonstrated the most inhibitory for Gram-positive *S. aureus*. 3 wt% chitosan showed the most antimicrobial activity on the microbial characteristics of Gouda cheese.

**Table 6.** Antimicrobial activity of investigated chitosan films

**Microorganisms**	**Chitosan**		

	**1 wt%**	**3 wt%**	**5 wt%**

	**Inhibition zone diameter (mm)**		
*E. coli*	25 ± 0.22^e^	39 ± 0.12^b^	21 ± 0.41^f^
*S. enterica*	26 ± 0.15^d^	39 ± 0.34^b^	15 ± 0.61^a^
*S. aureus*	39 ± 0.23^b^	49 ± 0.25^a^	28 ± 0.11^c^
*A. brasiliensis*	12 ± 0.31^i^	15 ± 0.13^h^	12 ± 0.15^i^
*S. cerevisiae*	11 ± 0.28^j^	16 ± 0.35^g^	11 ± 0.18^j^

The results were reported as mean ± SD. The different letters in each column indicate significant differences.

### The effects of chitosan-based nanocomposites concentration films on antimicrobial activity.

The results in Table 7 show the antimicrobial activity of nanocomposites made from chitosan concentrations by adding 1, 3 and 5 wt% nanoclay. Films with different concentrations have a significant inhibitory effect on all microorganisms. The highest growth inhibitory effect was observed in *S. aureus* and *E. coli*, respectively. The best antimicrobial activity on the microbial characteristics of Gouda cheese belongs to nanocomposite made from 3 wt% chitosan by adding 1 wt% nanoclay.

**Table 7.** Antimicrobial activity of investigated chitosan based nanocomposites

**Microorganisms**	**Samples of chitosan based nanocomposites (wt%)**								

	**1C:1N**	**1C:3N**	**1C:5N**	**3C:1N**	**3C:3N**	**3C:5N**	**5C:1N**	**5C:3N**	**5C:5N**

	**Inhibition zone diameter (mm)**								
*E. coli*	36 ± 0.67^f^	33 ± 0.32^h^	33 ± 0.53^h^	39 ± 0.29^d^	32 ± 0.46^i^	31 ± 0.25^j^	22 ± 0.28^e^	20 ± 0.11^q^	19 ± 0.27^r^
*S. enterica*	35 ± 0.23^e^	30 ± 0.14^e^	28 ± 0.45^k^	40 ± 0.11^c^	33 ± 0.24^h^	33 ± 0.61^h^	22 ± 0.21^o^	21 ± 0.2^p^	17 ± 0.39^s^
*S. aureus*	41 ± 0.21^b^	35 ± 0.36^g^	32 ± 0.32^i^	52 ± 0.15^a^	37 ± 0.36^e^	36 ± 0.57^f^	24 ± 0.39^m^	25 ± 0.44^l^	20 ± 0.43^q^
*A. brasiliensis*	20 ± 0.37^q^	15 ± 0.85^e^	14 ± 0.16^e^	25 ± 0.55^l^	20 ± 0.19^q^	19 ± 0.15^r^	14 ± 0.52^u^	13 ± 0.63^v^	11 ± 0.52^w^
*S. cerevisiae*	20 ± 0.52^q^	17 ± 0.17^s^	12 ± 0.22^d^	23 ± 0.36^n^	22 ± 0.1^e^	19 ± 0.25^r^	15 ± 0.15^t^	15 ± 0.58^s^	11 ± 0.31^w^

The results were reported as mean ± SD. The different letters in each column indicate significant differences.

C: Chitosan

N: Nanoclay

### The effects of nanoclay-based nanocomposites concentration films on antimicrobial activity.

Table 8 is demonstrated results of the antimicrobial activity of nanocomposites made from nanoclay concentrations by adding 1, 3 and 5 wt% chitosan. All samples demostrated the most inhibitory zone diameter for Gram-negative bacteria and *S. aureus.* 1 wt% nanoclay and 3 wt% chitosan nanocomposite had the most antimicrobial activity on the microbial characteristics of Gouda cheese. Also, 1 wt% nanoclay and 1 wt% chitosan nanocomposite showed similar performance with 1 wt% nanoclay and 3 wt% chitosan nanocomposite.

**Table 8.** Antimicrobial activity of investigated nanoclay based nanocomposites

**Microorganisms**	**Samples of nanoclay based nanocomposites (wt%)**								

	**1N:1C**	**1N:3C**	**1N:5C**	**3N:1C**	**3N:3C**	**3N:5C**	**5N:1C**	**5N:3C**	**5N:5C**

	**Inhibition zone diameter (mm)**								
*E. coli*	28 ± 0.67^f^	32 ± 0.67^e^	25 ± 0.67^l^	29 ± 0.67^h^	27 ± 0.67^j^	24 ± 0.67^m^	24 ± 0.67^m^	22 ± 0.67^n^	20 ± 0.67^p^
*S. enterica*	38 ± 0.67^a^	38 ± 0.67^a^	32 ± 0.67^e^	36 ± 0.67^c^	37 ± 0.67^b^	29 ± 0.67^h^	28 ± 0.67^i^	24 ± 0.67^m^	21 ± 0.67^o^
*S. aureus*	30 ± 0.67^g^	35 ± 0.67^d^	24 ± 0.6^m^	28 ± 0.67^i^	31 ± 0.67^f^	25 ± 0.67^l^	26 ± 0.67^k^	21 ± 0.67^o^	19 ± 0.67^q^
*A. brasiliensis*	14 ± 0.67^t^	16 ± 0.67^r^	13 ± 0.67^u^	11 ± 0.6^w^	13 ± 0.67^u^	12 ± 0.67^v^	12 ± 0.67^v^	11 ± 0.67^w^	0 ± 0.67^z^
*S. cerevisiae*	14 ± 0.67^t^	15 ± 0.67^s^	12 ± 0.67^v^	12 ± 0.67^v^	12 ± 0.67^v^	11 ± 0.6^w^	11 ± 0.67^w^	11 ± 0.67^w^	11 ± 0.6^w^

The results were reported as mean ± SD. The different letters in each column indicate significant differences.

C: Chitosan

N: Nanoclay

## DISCUSSION

The present study investigated the antimicrobial activity of Gouda cheese coating using chitosan, nanoclay and their nanocomposites.

It is demonstrated that the antimicrobial activity of chitosan was much greater than nanoclay, in agreement with similar study that reported the same results (21). Chitosan affected growth of all tested microorganisms including Gram-positive and Gram-negative bacteria, mold and yeast indicators. Also, it demonstrated the most inhibitory effects on *S. aureus*. Similarly, other studies have reported the same antibacterial activity of chitosan (19, 21). In agreement with findings in this study, Rejane C. Goy et al. and Chiu and et al. reported that chitosan is consistently more active against the Gram-positive *S. aureus* than Gram-negative *E. coli* (21, 23). In contrast, almost none of the nanoclay concentrations showed antimicrobial effects on mold and yeast and demonstrated the most inhibition on Gram-negative bacteria growth. Abdollahi et al. reported there no antibacterial activity in nanoclay (22).

In other words, adding nanoclay into chitosan and adding chitosan into nanoclay increased antimicrobial properties in nanocomposite. Therefore, adding nanoclay into chitosan film has more antimicrobial effect than chitosan film (20, 21).

The test results showed that when chitosan is located in the core of the nanocomposite and then nanoclay is added to it, the inhibitory effect will be greater. The best antimicrobial activity on the microbial limitations of Gouda cheese belongs to nanocomposite made from 3 wt% chitosan by adding 1 wt% nanoclay. The minimum antimicrobial activity of various films was observed in examined Mold and Yeast, which requires more investigation to improve its inhibition.

There is a possibility of contamination Gouda cheese during production, storage, transportation, delivery and time of consumption (24). The chitosan and nanoclay nanocomposite can also be used in similar semi-hard cheeses such as Adam cheese. It may also be used to coating fruit surfaces and food primary packaging to prevent microorganisms from infiltrating and growing to increase their shelf life and safety of food.

It is worth mentioning that further research recommended to investigate the chemical and physical properties of this type of coating and its effect on Gouda cheese.
